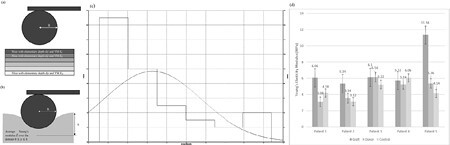# 533 Preliminary in Vivo and Atomic Force Microscopy Assessment of Uninjured Skin, Donor and Graft Sites

**DOI:** 10.1093/jbcr/irae036.167

**Published:** 2024-04-17

**Authors:** Apolline S Pistek, Lindsay Burnett, Vincent Gabriel, Shyla K Bharadia, Priyanka G Mukherjee, Mathias Amrein

**Affiliations:** University of Calgary, Calgary, AB; Alberta Health Services, Calgary, AB; Cumming School of Medicine University of Calgary, Calgary, AB; University of Calgary, Calgary, AB; Alberta Health Services, Calgary, AB; Cumming School of Medicine University of Calgary, Calgary, AB; University of Calgary, Calgary, AB; Alberta Health Services, Calgary, AB; Cumming School of Medicine University of Calgary, Calgary, AB; University of Calgary, Calgary, AB; Alberta Health Services, Calgary, AB; Cumming School of Medicine University of Calgary, Calgary, AB; University of Calgary, Calgary, AB; Alberta Health Services, Calgary, AB; Cumming School of Medicine University of Calgary, Calgary, AB; University of Calgary, Calgary, AB; Alberta Health Services, Calgary, AB; Cumming School of Medicine University of Calgary, Calgary, AB

## Abstract

**Introduction:**

With limited dermal function at the recipient site, fibrosis is expected following split-thickness skin grafting. Additional hypertrophic scar may develop within the graft, along seams and margins or at the donor site. This study focuses on the mechanical changes in the skin following split-thickness skin grafting as assessed by a non-invasive negative pressure device and atomic force microscopy to describe gross and sub-cellular mechanical properties in the differing skin states post-grafting.

**Methods:**

Participants with full-thickness burn injuries acutely managed with a split-thickness skin graft, closed donor and graft sites without any previous steroid injection or laser therapeutic interventions and a suitable control site were recruited. A negative pressure device was applied to grafted, donor, and uninjured contralateral control sites and response recorded to vacuum pressure and release to calculate Young’s elasticity modulus. A 4mm punch biopsy was harvested from each labelled site and immediately processed using atomic force microscopy using silicon cantilevers conjugated with a spherical glass tip (radius 2.5 μm;) in a custom 3D printed frame to determine Young’s elasticity based on the Hertz fit model (fig 1a-b).

**Results:**

A total of 5 participants (3 male, mean age 32.4 range 21-54, 152-427 days post-injury) have been recruited for the study thus far. Measurements were taken on the thigh of 4 participants and on the abdomen of 1. Atomic force microscopy data demonstrates lowest Young’s elasticity modulus (mean 1.49 ± 0.12 kPa) at the control sites (fig 1b graft 3: x-axis frequency, y-axis YM) with highest Young’s elasticity modulus (mean 2.70 ± 0.10 kPa) at the grafted sites. The donor sites are in the middle of the graft and control, (mean 1.98 ± 0.88 kPa). This trend is in concordance with clinical observation and elasticity probe measurement, where the graft is the toughest and the donor and control are similar (fig 1c).

**Conclusions:**

Initial elasticity measurement by a non-invasive vacuum device has been in agreement with atomic force microscopy assessment. Further sampling is required to verify preliminary results, simulate resting tension in the skin and assess change following intervention.

**Applicability of Research to Practice:**

Valid and reliable non-invasive measures will be informative of treatment effectiveness. Sub-cellular assessment of mechanical changes in skin graft and scar may direct therapeutic interventions.